# Bile Acid Profile Differs Between Brain Regions in Rodents and Is Disrupted in a Rodent Model of Alzheimer's Disease

**DOI:** 10.1002/cph4.70034

**Published:** 2025-08-04

**Authors:** Melanie A. Reuter, Rosalinda Moreno, Madelynn E. Agabao‐Tucker, Rahaf Shishani, Jessica Miranda Bustamante, Zara Marfori, Taylor Richieri, Anthony E. Valenzuela, Ameer Y. Taha, Pamela J. Lein, Renu Nandakumar, Bethany P. Cummings

**Affiliations:** ^1^ Department of Surgery Center for Alimentary and Metabolic Science, School of Medicine, University of California—Davis Sacramento California USA; ^2^ Department of Molecular Biosciences School of Veterinary Medicine, University of California—Davis Davis California USA; ^3^ Department of Food Science and Technology College of Agriculture and Environmental Sciences, University of California—Davis Davis California USA; ^4^ Biomarkers Core Laboratory Irving Institute for Clinical and Translational Research, Columbia University Irving Medical Center New York New York USA

**Keywords:** Alzheimer's disease, bile acids, gut–brain axis, liver–brain axis, resistant starch

## Abstract

Low but biologically relevant levels of bile acids are found in the brain and are altered in patients with Alzheimer's disease (AD). However, the regulation of brain bile acid levels and what drives brain bile acid dynamics are poorly understood. Bile acids are synthesized in the liver and further metabolized by bacteria in the gut. Therefore, bile acids are mediators of the liver–brain axis and the gut–brain axis. Additionally, whether the bile acid profile differs between brain regions and whether the brain region‐specific bile acid profile is impacted by disease, such as AD, is unknown. Therefore, we tested the hypothesis that the brain bile acid profile is influenced by peripheral bile acid metabolism, differs between brain regions, and that these dynamics change in AD. To this end, we assessed the bile acid profile in the cortex and hippocampus of wild‐type mice maintained on different diets. To test the effect of AD, we used the TgF344‐AD rat model. We found that the brain bile acid profile in mice was mildly altered by diet and, in both mice and rats, differs substantially between brain regions. For example, cholic acid and taurocholic acid are enriched in the cortex relative to the hippocampus in both mice and rats. Further, using a rat model of AD, we found that brain region differences in bile acid profiles are attenuated in AD. Together, these data demonstrate that both peripheral and central regulatory mechanisms maintain bile acid homeostasis in specific brain regions and that these homeostatic mechanisms are disrupted in AD.

## Graphical abstract

We find that bile acid profile differs substantially between brain regions in mice and rats. However, differences in bile acid profile between brain regions are attenuated in a rat model of Alzheimer's disease. Together, these data demonstrate that both peripheral and central regulatory mechanisms maintain brain bile acid homeostasis, which is disrupted in Alzheimer's disease.

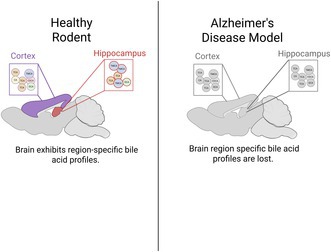

## 1 Introduction

Bile acids are diverse, amphipathic steroid molecules synthesized from cholesterol in the liver and further metabolized by bacteria in the gut. Small but biologically relevant levels of bile acids are found in both human and rodent brains (Ferdinandusse et al. 2009; Mertens et al. 2017). These molecules, classically known for their role in lipid digestion, are potent signaling molecules (Hylemon et al. 2009). The main bile acid receptors are Farnesoid X receptor (FXR) and Takeda G protein‐coupled receptor 5 (TGR5) (Hylemon et al. 2009; Chiang and Ferrell 2020; Holter et al. 2020). Bile acid signaling through these receptors exerts many physiologic effects, including regulation of hepatic bile acid metabolism, promotion of glucose homeostasis, and lowering of inflammatory cytokine secretion. These receptors are expressed and functional in the brain, and bile acid concentrations in the brain are sufficient to activate bile acid receptors (Kawamata et al. 2003; Maruyama et al. 2002; Huang et al. 2016; Keitel et al. 2010). For example, bile acid signaling through TGR5 in the brain has been found to decrease appetite and reduce neuroinflammation (Liang et al. 2021; Perino et al. 2021). While bile acids have a physiologically important role in the brain, the regulation of bile acid levels in the brain remains unclear.

The main pathway by which primary bile acids are produced is the classical pathway, which takes place in the liver and generates 90% of bile acids from cholesterol. The alternative pathway produces approximately 10% of bile acids and can be initiated in other tissues, including the brain (Duane and Javitt 1999; McMillin and DeMorrow 2016). Whether they are produced by the classical or alternative pathway, bile acids will undergo the final synthesis steps in the liver, where they are also conjugated to taurine or glycine (Chiang 2013). They then travel to the gallbladder, where they are stored. After a meal, bile acids are secreted into the small intestine, where they interact with bacteria. Gut bacteria perform a wide variety of bile acid modifications, including deconjugation of taurine or glycine through the action of bile salt hydrolase and 7α‐dehydroxylation. 7α‐dehydroxylation is the process by which the primary bile acids, cholic acid (CA) and chenodeoxycholic acid (CDCA), are converted into the secondary bile acids, deoxycholic acid (DCA) and lithocholic acid (LCA), respectively. Approximately 95% of bile acids are recycled via enterohepatic recirculation (Chiang 2013). Some bile acids can enter the systemic circulation, where they are distributed to tissues throughout the body, including the brain (Chiang 2013; Mano et al. 2004).

Conjugated and unconjugated primary and secondary bile acids are found in the brain (Baloni et al. 2020; MahmoudianDehkordi et al. 2019). However, the regulation of brain bile acid levels is poorly understood. Current literature suggests that the profile of bile acids in the brain is determined by peripheral bile acid metabolism, making bile acids key mediators of both the liver–brain and gut–brain communication (Xing et al. 2023; Higashi et al. 2017). Indeed, a prior study reported that bile acids enter the brain from the periphery via passive diffusion (Higashi et al. 2017). Further, the presence of secondary bile acids in the brain indicates a role for the gut microbiome in regulating the brain bile acid profile since secondary bile acids can only be synthesized by bacteria in the gut. However, the brain expresses some enzymes involved in primary bile acid metabolism (Baloni et al. 2020), specifically those involved in the alternative pathway of bile acid metabolism and the neural cholesterol clearance pathway. Both pathways generate bile acid precursors.

Alterations in bile acid profiles impact bile acid receptor signaling because bile acid subtypes have different affinities for bile acid receptors. For example, the secondary bile acids LCA and DCA have the highest affinity for TGR5, while primary bile acids have the highest affinity for FXR. Differing bile acid affinities for each receptor cause a diversity in host response as bile acid profiles change. Further, alterations in brain bile acid profiles have been implicated in neurological diseases. For example, bile acid levels in the dorsolateral prefrontal cortex are elevated in Alzheimer's disease (AD) compared to neurocognitively normal controls (Baloni et al. 2020; MahmoudianDehkordi et al. 2019; Pan et al. 2017).

We have previously shown that dietary resistant starch (RS) supplementation increases gut luminal abundance of DCA, a secondary bile acid, compared to mice receiving an isocaloric control diet (Reuter et al. 2024). This dietary probe caused robust changes in gut luminal bile acid profiles and shifts in every taxon of bacteria within the gut (Reuter et al. 2024). It is unknown if a diet that modulates the bile acid profile in the periphery can affect the bile acid profile in the brain. It is presumed that bile acid profiles in the brain are dictated based solely on bile acid diffusion from the periphery. Additionally, it is unknown if bile acid profiles differ between brain regions. AD has region‐specific pathophysiology, causing hallmark effects in the entorhinal cortex and hippocampus. While bile acid levels in the brain are found to be altered in AD, whether these changes are region‐specific has not been established. Therefore, we tested the hypothesis that the brain bile acid profile is influenced by peripheral bile acid metabolism, differs between brain regions, and that these dynamics change in AD. We focused on the bile acid profile in the hippocampus and mediotemporal cortex (in the region of the entorhinal cortex), as these are key brain regions impacted in AD. Herein, we report that diet mildly altered the brain bile acid profile, which differed significantly and consistently between brain regions, and these regional differences were lost in a rat model of AD. These data suggest that, in addition to peripheral inputs from the liver and gut, the healthy brain may regulate bile acid profiles locally and that this local regulation is compromised in AD.

## 2 Materials and Methods

### 2.1 Animal Studies

The Guide for the Care and Use of Laboratory Animals of the National Institutes of Health was followed for all animal studies. The experimental protocols were approved by the Institutional Animal Care and Use Committee of the University of California at Davis. All animals were housed in a temperature and humidity‐controlled room with a 12:12 h light–dark cycle. Male and female wild‐type C57BL/J mice were put on the isocaloric diet (IC) (Research Diets Inc.; diet number D21102808M) at 2 months of age for 2 months. Mice were then enrolled to continue the IC or RS‐supplemented diet (Research Diets Inc.; diet number D21102809M). Mice were assigned non‐identifying animal IDs and randomly assigned to groups, which were age, sex, and weight‐matched at the time of enrollment. Dietary intervention lasted for 1 (*n* = 4 per sex per group) or 2 months (*n* = 11–12 per sex per group). An equal amount of energy came from each macronutrient for each diet: 12.3% protein, 45.8% carbohydrate, and 40.1% fat, with an equal number of calories by weight: 4085 kcal/kg. Full details regarding the diet are available in our previously published work (Reuter et al. 2024). Measures of body weight and food intake were taken twice weekly. Mice were euthanized with an overdose of pentobarbital (200 mg/kg IP). Adipose depots were dissected and measured. The brain was removed and further dissected into the hippocampus and cortex then was flash frozen in liquid nitrogen before being stored at −80°C until analysis. Hippocampus and cortex of mice maintained on diets for 2 months were pooled such that bile acid analysis represents the brains of two‐three mice. Otherwise, each datapoint represents individual rodents. Male TgF344‐AD and wild‐type littermate rats were euthanized at 16 months of age for collection of serum and brain samples for bile acid profiling (*n* = 7 per group, and one rat was excluded as an outlier). Brains were perfused as previously described (Patten et al. 2021). Additionally, brain was perfused and collected from 10‐month‐old male TgF344‐AD and wild‐type rats for mRNA expression experiments (*n* = 3–5 per group).

### 2.2 Bile Acid Quantification

Hippocampus and cortex were analyzed for bile acids by UPLC‐MS/MS at the Columbia Biomarkers Core Laboratory in the Irving Institute for Clinical and Translational Research at Columbia University Medical Center as previously described using a Waters Xevo TQS mass spectrometer integrated with an Acquity UPLC system (Milford, MA) (Reuter et al. 2024). The 12‐OH/non‐12‐OH ratio was calculated as follows:

$$\text{Ratio}=\frac{\left[\mathrm{CA}\right]+\left[\mathrm{TCA}\right]+\left[\mathrm{GCA}\right]+\left[\mathrm{DCA}\right]+\left[\text{TDCA}\right]+\left[\text{GDCA}\right]}{\left[\text{Total bile acids}\right]-\left(\left[\mathrm{CA}\right]+\left[\mathrm{TCA}\right]+\left[\mathrm{GCA}\right]+\left[\mathrm{DCA}\right]+\left[\text{TDCA}\right]+[\text{GDCA]}\right)}$$

### 2.3 qPCR

qPCR was performed on cortex from mice after 2 months of dietary intervention, as previously described (Reuter et al. 2024). RNA was extracted using the guanidine thiocyanate method and RNA spin columns (Epoch Life Sciences; Cat# 1940‐050). cDNA was synthesized as per manufacturer instructions using the Bio‐Rad iScript cDNA Synthesis Kit. qPCR was performed using the Sso Advanced Universal SYBR Green Mastermix (Bio‐Rad Laboratories Inc.; Catalog number: 1725272) according to manufacturer instructions. Plates were read using the Applied Biosystems StepOnePlus Real‐Time PCR System (Applied Biosystems; Cat# 4376592). Primers were designed using the IDT PrimerQuest tool and the Basic Local Alignment Search Tool (BLAST). Primer sequences are available in Table S1. Data were analyzed using the 2^−ΔΔCT^ method.

### 2.4 Data Analysis and Statistics

Data are presented as mean ± SEM. All statistical analyses were performed using GraphPad Prism 10.1.2 for Windows (GraphPad Software, San Diego, CA, USA). Data were analyzed using unpaired two‐tailed Student's *t*‐test, Mann–Whitney test, two‐stage step‐up Mann–Whitney tests, two‐factor repeated measures ANOVA with Bonferroni posttest, or chi‐squared, as appropriate. All data were assessed for normality; if data were normally distributed, Student's *t*‐test or ANOVA were used, depending on the number of groups. If data were not normally distributed, Mann–Whitney or two‐stage step‐up Mann–Whitney tests were used. Differences were considered significant at *p* < 0.05. When correcting for multiple comparisons in the two‐stage step‐up Mann–Whitney test, a false discovery rate of *Q* = 0.05 was used. Bile acid subtypes were considered detectable if more than half of the samples had quantifiable readings in either group. Outliers were excluded using Grubb's test *α* = 0.01.

## 3 Results

### 3.1 Resistant Starch Supplementation Produces Only Subtle Changes in Brain Bile Acid Profile

**FIGURE 1:**
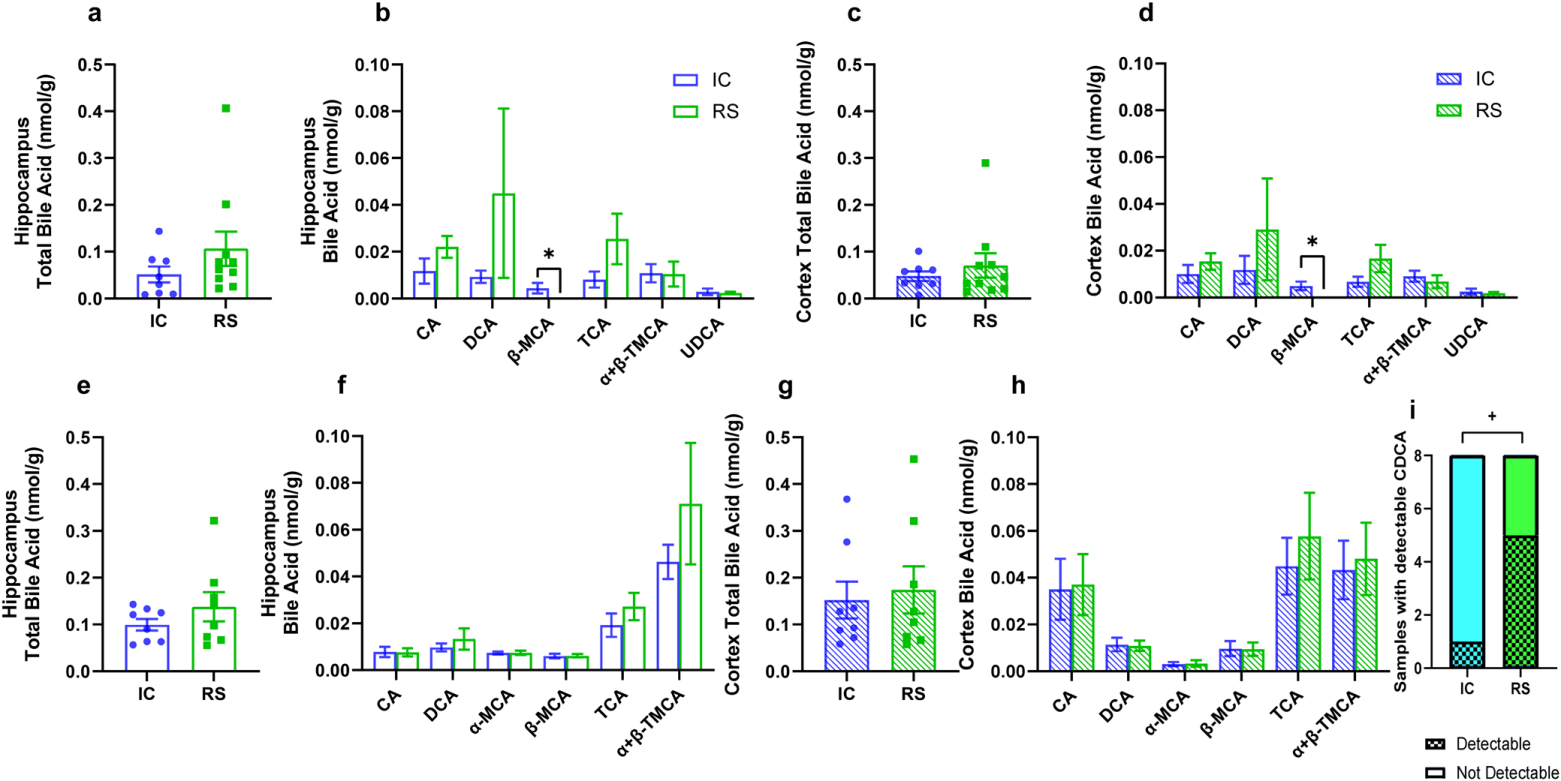
Bile acid profile in the brain changes subtly with diet. (a) Total bile acid concentration in the hippocampus and (b) bile acid subtype concentrations in the hippocampus after 1 month of IC or RS dietary intervention. (c) Total bile acid concentration in the cortex and (d) bile acid subtype concentrations in the cortex after 1 month of IC or RS dietary intervention. (e) Total bile acid concentration in the hippocampus and (f) bile acid subtype concentrations in the hippocampus after 2 months of IC or RS dietary intervention. (g) Total bile acid concentration in the cortex, (h) bile acid subtype concentrations in the cortex, and (i) number of brain samples with detectable CDCA after 2 months of IC or RS dietary intervention. Each dot represents a data point from a single mouse in panels a–d and a data point generated from samples pooled from two‐three mice in panels e–h. Data are presented as mean ± SEM. *p < 0.05 by two‐stage step‐up Mann–Whitney test, ^+^ *p* < 0.05 by chi‐squared test. *n* = 8 per group.

We previously found that feeding mice a diet supplemented with RS increases DCA abundance in gut luminal contents compared to mice receiving an IC control diet (Reuter et al. 2024). Using samples from the same mice that we previously reported on (Reuter et al. 2024), we assessed the bile acid profile in the hippocampus and cortex of mice that were maintained on the IC diet for 2 months and were then divided into a group that continued on the IC diet or switched to the RS diet for 1 month prior to euthanasia and sample collection. The total bile acid concentration did not differ between groups in the hippocampus (Figure 1a). The concentration of β‐MCA in the hippocampus was lower in RS compared with IC‐fed mice (Figure 1b, *p* < 0.05). Similarly, total bile acid concentration in the cortex did not differ between groups (Figure 1c), and the concentration of β‐MCA in the cortex was lower in RS compared with IC‐fed mice (Figure 1d, *p* < 0.05).

To determine if a longer dietary exposure induced different effects on the brain bile acid profile, the study was repeated, and samples were collected after 2 months of dietary intervention. Further, to determine if we could detect additional bile acid subtypes by analyzing a larger amount of tissue while maintaining brain region‐specific outcomes, we pooled brain regions from two‐three mice per sample to increase the sample amount used for analysis. Similar to our previous work (Reuter et al. 2024), there was no difference in body weight, food intake, or adiposity between mice receiving the IC compared with the RS supplemented diet for 2 months (Figure S1a–g). Increasing the sample amount analyzed by pooling brain samples together allowed us to detect α‐muricholic acid. Total bile acid concentration and the concentration of individual bile acids did not differ between groups in the hippocampus (Figure 1e,f) or cortex (Figure 1g,h). However, RS supplementation increased the frequency of detection of CDCA in the brain, compared to IC‐fed control mice (Figure 1i, *p* < 0.05).

To investigate whether the diet may have altered the expression of enzymes involved in bile acid metabolism in the brain, qPCR was performed on cortex tissue from mice after 2 months of IC or RS intervention. CDCA precursors are made in the brain as part of the alternative pathway of bile acid metabolism and the neural cholesterol clearance pathway (Petrov et al. 2016). *Cyp27a1* and *Cyp7b1* are key enzymes in the alternative pathway (Petrov et al. 2016; Kakiyama et al. 2019). *Cyp46a1* and *Cyp39a1* are key enzymes in the neural cholesterol clearance pathway (Petrov et al. 2016). Bile acids are transported primarily by the transporter *Asbt*. There was no difference between groups in mRNA expression *of Cyp27a1, Cyp39a1, Cyp46a1, Cyp7b1*, or *Asbt* in the cortex (Figure S2a–e). Together, these data show that diet‐induced changes in peripheral bile acid metabolism decrease concentrations of β‐MCA in the brain after 1 month of dietary intervention and increase CDCA in the brain after 2 months of dietary intervention.

### 3.2 The Brain Exhibits Region‐Specific Bile Acid Profiles

**FIGURE 2:**
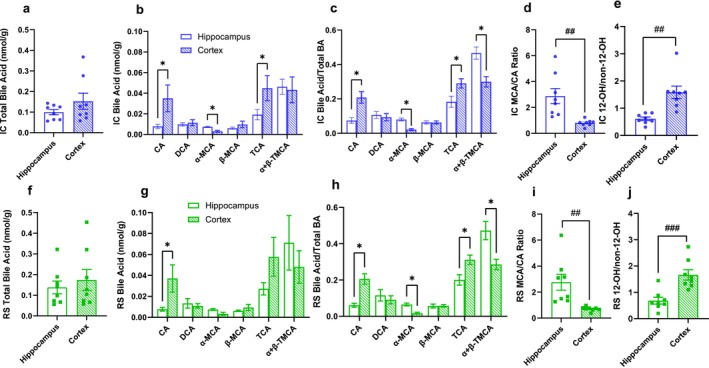
The mouse brain exhibits brain region‐specific bile acid profiles. (a) Total bile acid concentration, (b) bile acid subtype concentrations, (c) bile acid subtype proportions, (d) MCA/CA ratio, and (e) 12OH/non‐12OH ratio after 2 months of IC dietary intervention in the hippocampus and cortex. (f) Total bile acid concentration, (g) bile acid subtype concentrations, (h) bile acid subtype proportions, (i) MCA/CA ratio, and (j) 12OH/non‐12OH ratio after 2 months of RS dietary intervention in the hippocampus and cortex. Each dot represents a data point generated from samples pooled from two‐three mice. Data are presented as mean ± SEM, *n* = 8 per group. *p < 0.05 by two‐stage step‐up Mann–Whitney test, ^##^ *p* < 0.01, ^###^ *p* < 0.001 by Student's *t*‐test.

Next, we assessed whether the bile acid profile differed between brain regions. For this analysis, we focused on the dataset generated from the pooled brain region samples collected from mice after 2 months of IC or RS diet intervention (Figure 1e–i). Total bile acid concentrations did not differ between the hippocampus and cortex of IC‐fed mice (Figure 2a). CA and TCA concentrations were higher in the cortex, while α‐MCA concentrations were lower in the cortex compared to the hippocampus (Figure 2b, *p* < 0.05). CA and TCA expressed as a proportion of total bile acids were higher in the cortex compared to the hippocampus of IC‐fed mice (Figure 2c, *p* < 0.05). α‐MCA and α + β‐TMCA expressed as a proportion of total bile acids were lower in the cortex compared to the hippocampus of IC‐fed mice (Figure 2c, *p* < 0.05).

Interestingly, the MCA/CA ratio was lower in the cortex compared to the hippocampus of IC‐fed mice (Figure 2d, *p* < 0.01). The MCA/CA ratio gives information about hydrophobicity, as MCA is a highly hydrophilic bile acid subtype (Heuman 1989). Similar to the MCA/CA ratio, the 12OH/non‐12OH ratio measures the ratio of CA and its downstream products to CDCA and all its downstream products, including MCA. Consistent with the MCA/CA ratio, the 12OH/non‐12OH ratio was higher in the cortex compared to the hippocampus in IC‐fed mice (Figure 2e, *p* < 0.01). Just as in the IC‐fed mice, there was no change in the total bile acids between the cortex and hippocampus in the RS‐fed mice (Figure 2f). RS‐fed mice had higher levels of CA in the cortex compared to the hippocampus (Figure 2g, *p* < 0.05). The same changes in the relative abundance of bile acids found in the IC‐fed mice were present in the RS‐fed mice, with an increased abundance of CA and TCA and a decreased abundance of α + β‐TMCA and α‐MCA in the cortex compared to the hippocampus (Figure 2h, *p* < 0.05). Similar to the IC‐fed mice, the RS‐fed mice had a lower MCA/CA ratio and a higher 12OH/non‐12OH ratio in the cortex compared with the hippocampus (Figure 2i,j, *p* < 0.01). Together, these data highlight important differences in bile acid profile between brain regions.

### 3.3 Brain Bile Acid Levels Are Elevated in a Rat Model of Alzheimer's Disease

Given our findings in wild‐type mice that bile acid profiles differ between brain regions, we next determined whether brain region‐specific bile acid profiles are impacted in disease. We focused on AD, given recent studies that bile acid profiles are altered in the serum and brain of patients with AD (Baloni et al. 2020; MahmoudianDehkordi et al. 2019; Pan et al. 2017; Marksteiner et al. 2018). We studied the TgF344‐AD rat model of AD (hereafter referred to as TG), which develops amyloid beta plaques and tauopathies similar to those found in human AD patients (Cohen et al. 2013), and thus, it has been leveraged by numerous laboratories to study the physiological drivers for memory loss associated with AD (Bac et al. 2023; Fowler et al. 2022; Bernaud et al. 2022). We collected serum, hippocampus, and cortex from 16‐month‐old wild‐type (WT) and TgF344‐AD (TG) littermates to determine the effect of AD on brain versus peripheral bile acid profiles.

**FIGURE 3:**
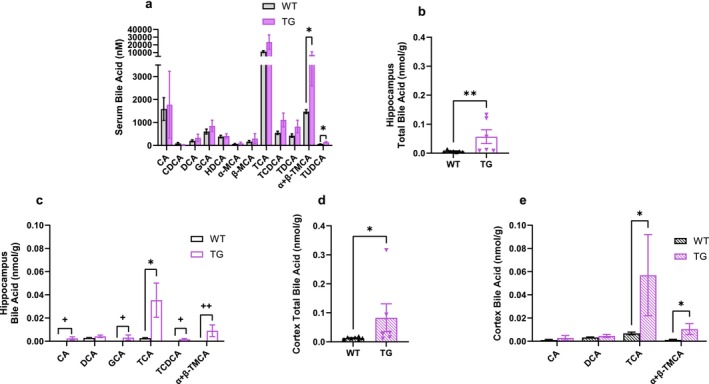
Brain bile acid concentrations are higher in a rat model of AD relative to wild‐type controls. (a) Serum bile acid subtype concentrations, (b) total bile acid concentration in the hippocampus, (c) bile acid subtype concentrations in the hippocampus, (d) total bile acid concentration in the cortex, and (e) bile subtype concentrations in the cortex in 16‐month‐old WT and TG rats. Each dot represents a data point from a single rat. Data are presented as mean ± SEM. *n* = 6–7 per group. *p < 0.05 and **p < 0.01 by Mann‐Whitney test or two‐stage step‐up Mann–Whitney test. ^+^ *p* < 0.05, and ^++^ *p* < 0.01 by chi‐squared test.

α + β‐TMCA and TUDCA serum concentrations were elevated in TG compared to WT rats (Figure 3a, *p* < 0.05). Total bile acid concentration in the hippocampus was higher in TG compared to WT rats (Figure 3b, *p* < 0.01). Further, there were more detectable bile acid subtypes in the hippocampus of TG rats compared to WT. Specifically, the concentration of TCA, CA, GCA, TCDCA, and α + β‐TMCA in the hippocampus was higher in TG compared to WT rats (Figure 3c, *p* < 0.05). Total bile acid concentrations were higher in the cortex of TG compared with WT rats (Figure 3d *p* < 0.05). As in the hippocampus, TCA and α + β‐TMCA concentrations in the cortex were higher in TG compared to the WT rats (Figure 3e, *p* < 0.05). These data are consistent with a previous study of human samples that reported an increase in TCA concentration in the dorsolateral prefrontal cortex of individuals with AD compared to neurocognitively normal controls (Baloni et al. 2020). Bile acid concentrations in the brain are well documented to be lower than in the serum, but brain bile acid profiles are thought to reflect the profile in the serum (Pan et al. 2017). While the increase in serum α + β‐TMCA concentrations in TG compared with WT rats was reflected in both the hippocampus and cortex, the increase in hippocampus and cortex TCA concentration in TG compared to WT rats was not reflected in the serum, suggesting that processes other than passive diffusion from serum to brain may be involved in the determination of brain bile acid profile.

### 3.4 Brain Region‐Specific Bile Acid Profile Is Lost in a Rat Model of Alzheimer's Disease

**FIGURE 4:**
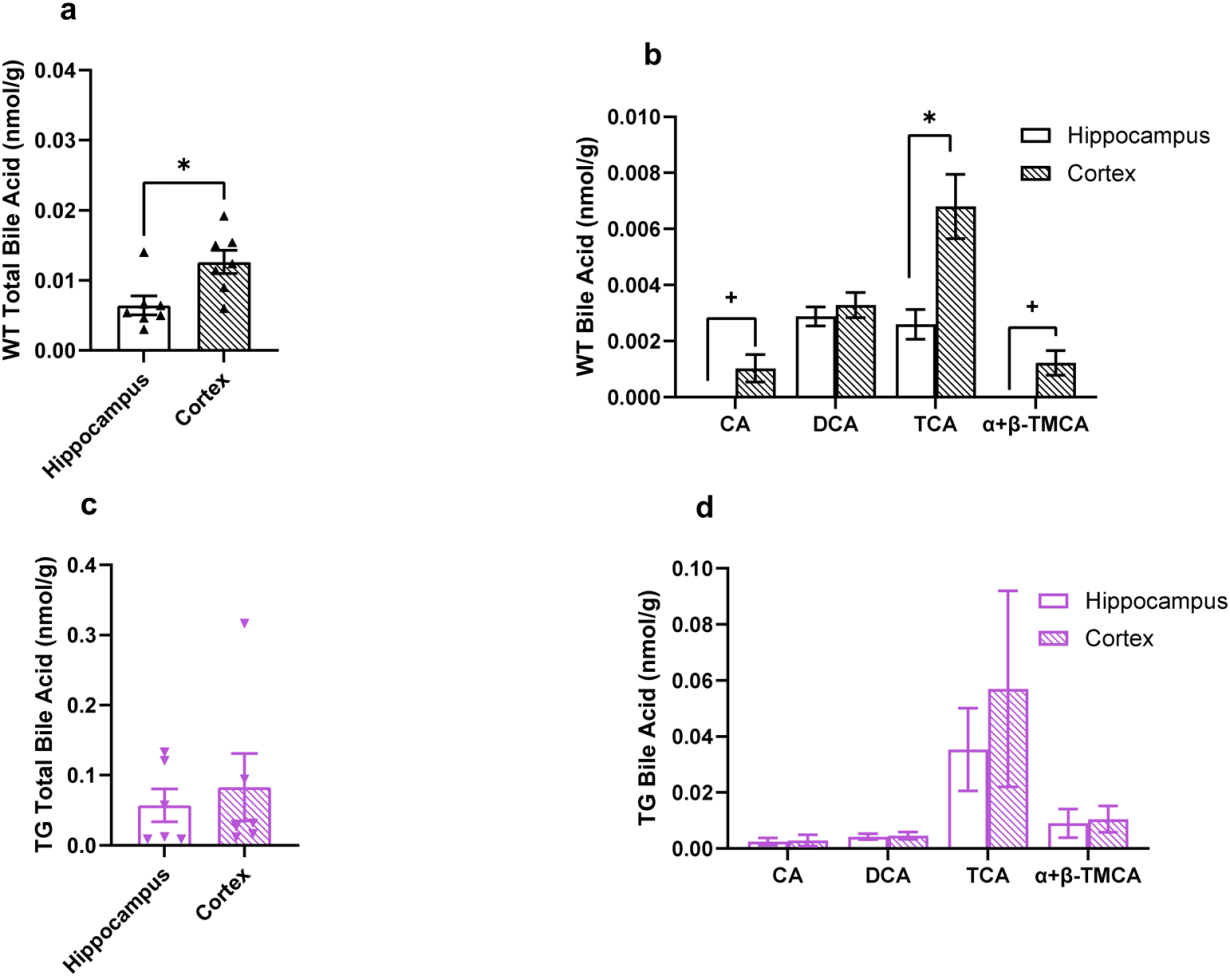
Brain region‐specific bile acid profile in lost in a rat model of AD relative to wild‐type controls. (a) Total bile acid concentration and (b) bile acid subtype concentrations in the hippocampus and cortex of 16‐month‐old wild‐type rats. (c) Total bile acid concentration and (d) bile acid sub‐type concentrations in the hippocampus and cortex of 16‐month‐old TG rats. *n* = 6–7 per group. Each dot represents a data point generated from a single rat. Data are presented as mean ± SEM. *p < 0.05 by Mann‐Whitney test or two‐stage step‐up Mann–Whitney test. ^+^ *p* < 0.05 by chi‐squared test.

Next, we determined the impact of genotype on brain region‐specific bile acid profiles to determine if this is changed in AD. Total bile acid concentration in WT rats was higher in the cortex compared to the hippocampus (Figure 4a, *p* < 0.05). The WT rats had higher levels of TCA, CA, and α + β‐TMCA in the cortex compared to the hippocampus (Figure 4b, *p* < 0.05). CA and TCA concentrations were higher in the cortex compared with the hippocampus in WT rats (Figure 4b, *p* < 0.05). These data support our findings in mice that bile acid profile differs between brain regions. Interestingly, total bile acid concentration and bile acid profile did not differ between brain regions in TG rats (Figure 4c,d), demonstrating that brain region‐specific bile acid profile was lost in aged TG rats.

### 3.5 Bile Acid Precursor‐Producing Enzymes Exhibit Region‐Specific Expression Patterns

**FIGURE 5:**
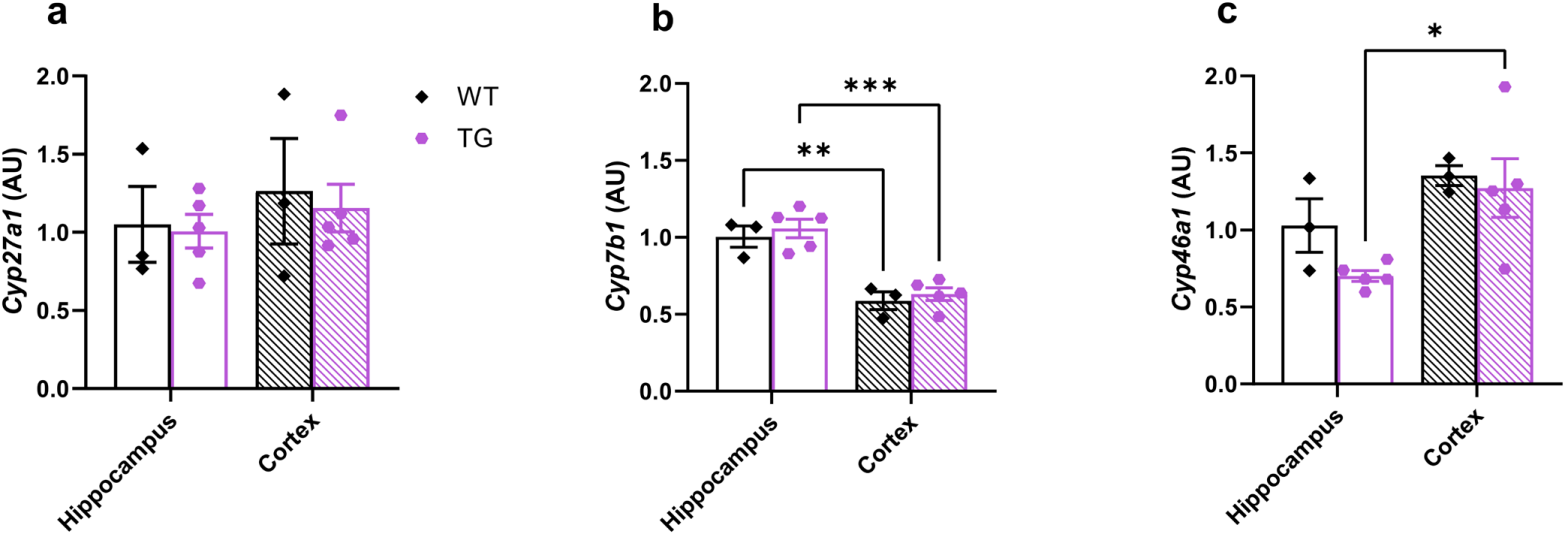
Brain region‐specific expression of bile acid producing enzymes. (a) *Cyp27a1*, (b) *Cyp7b1*, and (c) *Cyp46a1* mRNA expression in the hippocampus and cortex of 10‐month‐old TG and WT rats. *n* = 3–5 per group. Each dot represents a data point generated from a single rat. Data are presented as mean ± SEM *p < 0.05, **p < 0.01, and ***p < 0.001 by two‐factor ANOVA with Bonferroni posttest.

One potential mechanism contributing to regional differences in bile acid profiles in the brain is regional differences in expression of bile acid metabolizing enzymes. To evaluate this possibility, we assessed the expression of bile acid metabolizing enzymes *Cyp27a1, Cyp7b1*, and *Cyp46a1* in the cortex and hippocampus of WT and TG rats. *Cyp27a1* mRNA expression did not differ between genotypes or brain regions (Figure 5a). *Cyp7b1* mRNA expression was lower in the cortex compared to the hippocampus in both the WT and TG rats (Figure 5b, *p* < 0.01). Conversely, *Cyp46a1* mRNA expression was higher in the cortex compared to the hippocampus in TG rats only (Figure 5c, *p* < 0.05). Together, these data demonstrate that the expression of *Cyp7b1* and *Cyp46a1* differs between brain regions, and that for *Cyp46a1*, these differences depend on genotype. Differences in enzyme expression may contribute to the observed brain region‐specific differences in bile acid profile.

## 4 Discussion

While it is known that bile acids and their receptors are present in the brain, our understanding of the regulation of the brain bile acid profile has been limited. While bile acid concentrations in the brain are lower than elsewhere in the body, the concentrations present in the brain are sufficient to activate bile acid receptors (Kawamata et al. 2003; Maruyama et al. 2002). Herein, we provide new information regarding the assessment of bile acid profiles in response to diet across different brain regions. We find that dietary‐induced changes in peripheral bile acid metabolism cause mild changes to the brain bile acid profile and that healthy mice and rats exhibit distinct brain region‐specific bile acid profiles. Further, we find that the brain region‐specific bile acid profiles are lost in a rat model of AD. Prior work has suggested that the brain bile acid profile is determined by circulating bile acid levels (Xing et al. 2023; Higashi et al. 2017). Interestingly, our data indicate that region‐specific profiles are present, even in brain samples that have been perfused to remove blood. Therefore, our data suggest that the regulation of brain bile acid levels may be more nuanced and involve interactions between peripheral bile acid metabolism and local regulatory systems within the brain which become impaired in AD.

Brain region‐specific bile acid profiles differ in two ways: (1) differences in individual bile acid subtypes, and (2) differences in bile acid ratios that describe the overall composition of the bile acid pool. The cortex had higher levels of CA and TCA and lower levels of α‐MCA and TMCA than the hippocampus of mice fed the IC and RS diets. Higher TMCA in the hippocampus is consistent with findings that β‐TMCA accumulates in the hippocampus of mice as they age (Ma et al. 2024). The CA and MCA molecules differ at positions 6 and 12 of the steroid backbone. As a result, MCA is much more hydrophilic than CA. Therefore, different bile acid subtypes may end up in the hippocampus or cortex due to hydrophobicity preferences or regional differences in blood–brain barrier (BBB) permeability. Other possible explanations include region‐specific expression of bile acid transporters and enzymes. Several different bile acid transporters have been reported to be expressed in the brain, though their expression levels are low (Mertens et al. 2017; McMillin and DeMorrow 2016; Choudhuri et al. 2003; Seward et al. 2003; Ose et al. 2010). Almost all differences in individual bile acid subtypes by brain region found in mice were observed in the WT rats. This strong agreement between species could imply that mechanism(s) of regional specificity are conserved. The loss of brain region specificity in the bile acid profiles of the TG rats points to an impaired ability for the brain to regulate its bile acid profile in AD.

Bile acid ratios are also often used to generally describe the bile acid profile. One such ratio is the MCA/CA ratio, which can be used to describe the hydrophobicity of the bile acid pool. In the mouse brain, we observe that the MCA/CA ratio is lower in the cortex compared to the hippocampus in both IC and RS fed mice, indicating a more hydrophilic bile acid profile in the hippocampus. Another common ratio used to describe the bile acid pool is the 12OH/non‐12OH ratio. We observed a higher 12OH/non‐12OH ratio in the cortex than the hippocampus of both IC and RS fed mice. This ratio is generally used to describe the activity of the enzyme CYP8B1, which hydroxylates at the 12α position on the sterol backbone during bile acid synthesis. Elevations in the 12OH/non‐12OH ratio have been associated with insulin resistance, obesity, and high fat diet feeding (Hori et al. 2020; Haeusler et al. 2013). The information that the 12OH/non‐12OH ratio can provide with respect to brain physiology remains to be seen.

One explanation for region‐specific bile acid profiles is region‐specific expression of bile acid precursor producing enzymes. We measured levels of *Cyp46a1*, the rate‐limiting step in the neural cholesterol clearance pathway, and *Cyp27a1* and *Cyp7b1*, the first two enzymes in the alternative pathway of bile acid production in the rat brain. Consistent with a previous autopsy‐based study finding that *Cyp46a1* expression is increased in the human cortex compared to the hippocampus (Popiolek et al. 2020), we found that *Cyp46a1* levels were increased in the cortex in the TG rats. We also found that *Cyp7b1* levels decreased in the cortex compared to the hippocampus in both the WT and TG rats, consistent with previous findings (Stapleton et al. 1995; Maehata et al. 2020).

The BBB regulates bile acid entry into the brain. Due to their hydrophobic nature, bile acids typically travel in the blood bound to albumin, but only free bile acids can cross the BBB. The bile acid subtype affinities for albumin are driven by hydrophobicity, as follows: LCA > CDCA > DCA > UDCA > CA > TUDCA > TCA (Heuman 1989). Unconjugated bile acids can cross membranes either by passive diffusion or through active transport, and conjugated bile acids need a transporter to cross membranes (Dawson et al. 2009). If passive diffusion were the only mechanism for bile acids to cross the BBB, unconjugated bile acids would be present in levels according to their albumin binding. However, our data show that DCA is always detectable in the brain, even in cases where UDCA or TUDCA are not. The presence of DCA in the brain is not based on the serum availability of bile acids, either. Comparing the 1‐month serum bile acid concentrations, which have been previously reported (Reuter et al. 2024), to the brain concentrations, CA, TCA, and β‐MCA are the predominant bile acids in the serum. However, in the brain, DCA is one of the most abundant bile acids. Similarly, in the rat serum, the most abundant bile acids are CA, TCA, and α + β‐TMCA. However, CA and α + β‐TMCA are lowly abundant in the brain, while DCA is always present. Together, our data indicate a preferential uptake for certain bile acid types over others into the brain.

Patients and rodent models of AD have altered serum bile acid profiles (MahmoudianDehkordi et al. 2019; Pan et al. 2017; Ehtezazi et al. 2023; Shao et al. 2020; Chen et al. 2024). In fact, serum bile acids have been suggested as a biomarker for AD (Marksteiner et al. 2018; Chen et al. 2024). Increased concentrations of bile acids have been observed in the serum of patients with AD (Marksteiner et al. 2018; Shao et al. 2020). Similarly, we find that serum TUDCA and α + β‐TMCA concentrations are higher in TgF344‐AD rats compared with wild‐type controls. This is consistent with previous studies that have established increased levels of conjugated bile acids with AD, cognitive decline, and decreased cortical thickness in humans (Nho et al. 2019; Ren et al. 2024). TUDCA is one of the most hydrophilic bile acid subtypes and is being investigated as a treatment for several neurodegenerative disorders, including AD (Arnold et al. 2024). In addition to altered serum profiles, people with AD have altered brain bile acid profiles (Baloni et al. 2020; MahmoudianDehkordi et al. 2019; Pan et al. 2017). Levels of taurine‐conjugated bile acids, specifically TCA, have been reported to be elevated in the brain tissue of patients with AD (Baloni et al. 2020; MahmoudianDehkordi et al. 2019). Similarly, we report elevated levels of multiple taurine‐conjugated bile acids, including TCA, in both the hippocampus and cortex of the rat model of AD compared to WT control rats.

Our understanding of the role of bile acid receptor signaling in AD pathophysiology is nascent. Bile acids may exert protective effects by signaling through TGR5. Enhanced TGR5 signaling has been shown to be neuroprotective in rodent models, at least in part, by reducing neuroinflammation (Liang et al. 2021; Mu et al. 2022; Wu et al. 2018, 2019). Few studies have assessed the impact of FXR on AD pathophysiology. One study reported that FXR ablation in mice impaired memory and reduced motor coordination (Huang et al. 2015). Another study using a cell culture model suggested that FXR aggravated Aβ‐triggered neuronal apoptosis (Chen et al. 2019). Additionally, in disease states and at high concentrations, bile acids can cause breakdown of the BBB (Quinn et al. 2014; Greenwood et al. 1991) Interestingly, TGR5 activation protects the BBB, indicating a potential for bile acids to attenuate their own effect on the BBB in healthy rodents (Liang et al. 2020). As noted in Figure 4, bile acid concentrations were higher in the rat model of AD. Therefore, it is possible that the loss of region specificity is caused by the bile acids themselves. Further work is needed to define the role of bile acids and bile acid receptor signaling in AD pathogenesis, as advancements in this area may lead to the development of new therapeutic strategies for AD treatment.

## 5 Conclusion

The normal rodent brain bile acid profile is impacted, at least to some extent, by dietary interventions aimed at altering peripheral bile acid metabolism. Healthy rodents exhibit brain region‐specific bile acid profiles that are disrupted in a rat model of AD. Similarly, bile acids are altered in the brain of people with AD, though understanding of the regulation of the brain bile acid profile and the role of bile acids in the pathogenesis of AD is limited. Our data expand our understanding of brain bile acid dynamics and suggest the utility of manipulating bile acids to prevent or slow the progression of AD.

## Author Contributions

M.A.R.: Investigation, formal analysis, visualization, and writing – original draft. M.E.A.‐T., R.S., J.M.B., T.R., Z.M., and R.M.: Investigation. R.N.: validation and investigation. A.E.V.: resources. A.Y.T., P.J.L.: Resources, and writing – review and editing. B.P.C. Conceptualization, designed the experiments, supervised the project, acquired funding, project administration, and writing – original draft. All authors have reviewed and edited the manuscript.

## Ethics Statement

The Guide for the Care and Use of Laboratory Animals of the National Institutes of Health was followed for all animal studies. The experimental protocols were approved by the Institutional Animal Care and Use Committee of the University of California at Davis.

## Conflicts of Interest

The authors declare no conflicts of interest.

## Supporting information


**Data S1:** Supplementary Figures.

## Data Availability

The data that support the findings of this study are available from the corresponding author upon reasonable request.
