# Supplementary material for: Bile Acid Profile Differs Between Brain Regions in Rodents and Is Disrupted in a Rodent Model of Alzheimer's Disease
Source: Compr Physiol. 2025 Aug 4;15(4):e70034. doi: 10.1002/cph4.70034 (PMC12320573; doi:10.1002/cph4.70034)
Supplement: Supplementary file 1 — Data S1: Supplementary Figures. [file CPH4-15-e70034-s001.pdf]

SUPPLEMENTAL INFORMATION TITLES AND LEGENDS

Supplemental Figure S1. RS supplementation did not alter body weight, food intake, or adiposity compared to the IC diet. a) Body weight and **b)** food intake of mice fed 2 months of RS or IC diet. These metrics were only measured for the first 2 groups of mice. *n*=8 per group. **c)** White adipose, **d)** mesenteric adipose, **e)** perigonadal adipose, **f)** retroperitoneal, and **g)** subcutaneous adipose tissue weight after 2 months of IC or RS diet. *n*=23-24 per group. Data are presented as mean ± SEM.

Supplemental Figure S2. RS supplementation did not alter bile acid enzyme expression in cortex. a) *Cyp27a1*, **b)** *Cyp7b1*, **c)** *Cyp46a1*, **d)** *Cyp39a1*, and **e)** *Asbt* expression the cortex of IC vs RS-fed mice. *n*=8 per group. Data are presented as mean ± SEM.

Supplemental Tables

Supplemental Table S1. Primers

Primer	Forward 5' → 3'	Reverse 5' → 3'
**Mouse *Cyp27a1***	GCCTCACCTATGGGATCTTCA	TCAAAGCCTGACGCAGATG
**Mouse *Cyp7b1***	GGCATGACGATCCTGAAATA	TACATTGCCCAGAACATAGC
**Mouse *Cyp46a1***	CAGCTTCCTTCTTGGACATC	CCATACTTCTTAGCCCAATCC
**Mouse *Cyp39a1***	CAGTTCCTTCTGGTGATCTG	TTCCAGCGTTCAGGTTTG
**Mouse *Asbt***	GGGGTATCTTCGTGGGCTTC	TGCTAACACTGAGGTCCATGTC
**Mouse *Gapdh***	CAATGTGTCCGTCGTGGA	GATGCCTGCTTCACCACC
**Rat *Cyp27a1***	GAGGAAGAGAGAGGACGATAA	CATCTCCAGTTCTGCAATCC
**Rat *Cyp7b1***	GCGGAAAGGAGACTTTGTAG	ACGAAGCGATCAAACCTAAA
**Rat *Cyp46a1***	GGATTGGGCTAAGAAGTATGG	TGGTGGACATCAGGAACT
**Rat *Cyp39a1***	CAGCCACACTCAATACTCTC	CCATACTCAAAGCCCTCATC
**Rat *Asbt***	AGGGTCTGAGCTTGACTATC	CTCCTGGCTTCTCCTTTCTA
**Rat *Actin***	TTGGCACCACACTTTCTAC	TTTCACGGTTGGCCTTAG

Figure S1

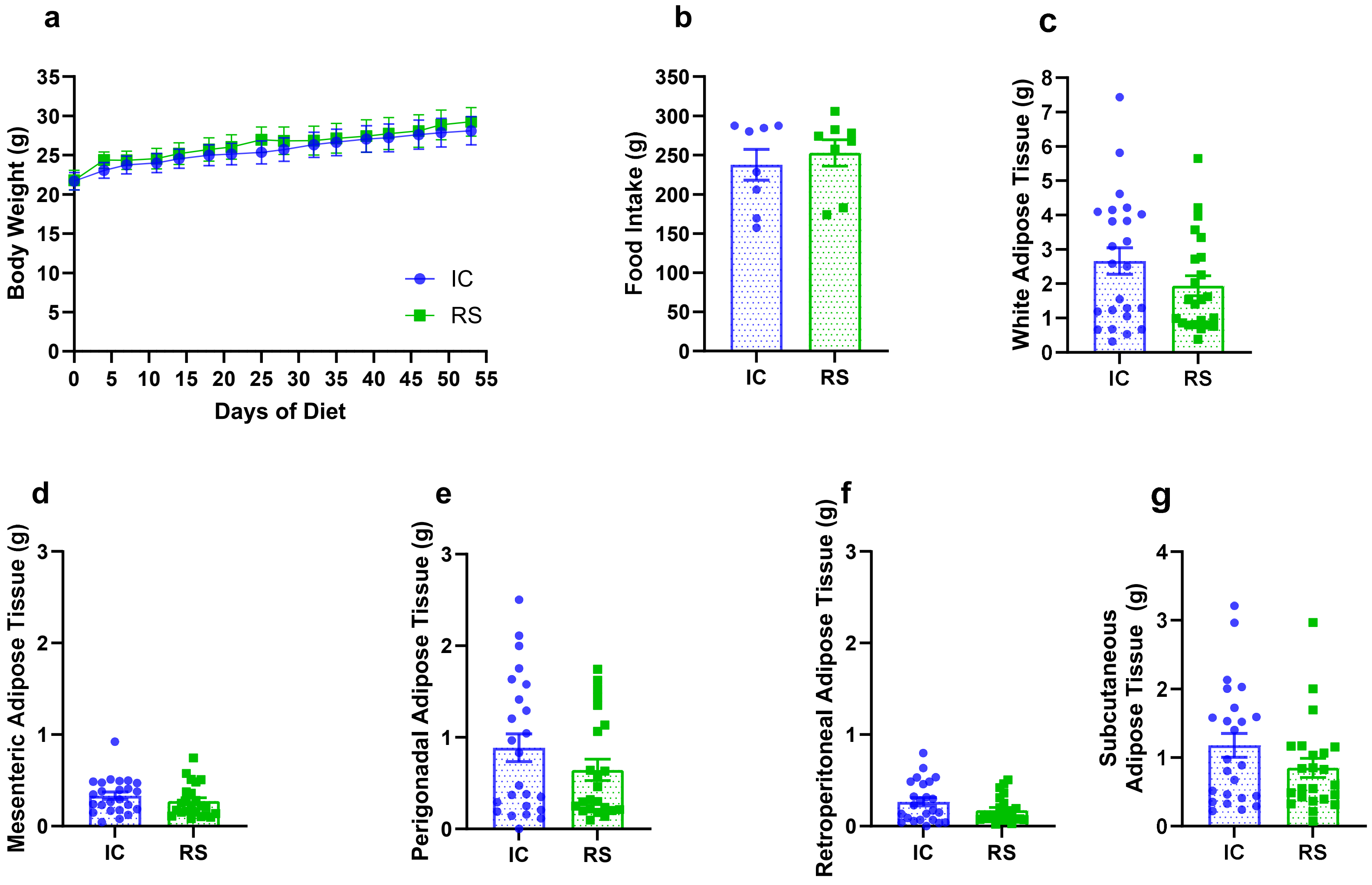

a
Body Weight (g)
Days of Diet
IC
RS
b
Food Intake (g)
c
White Adipose Tissue (g)
d
Mesenteric Adipose Tissue (g)
e
Perigonadal Adipose Tissue (g)
f
Retroperitoneal Adipose Tissue (g)
g
Subcutaneous Adipose Tissue (g)

Figure S2

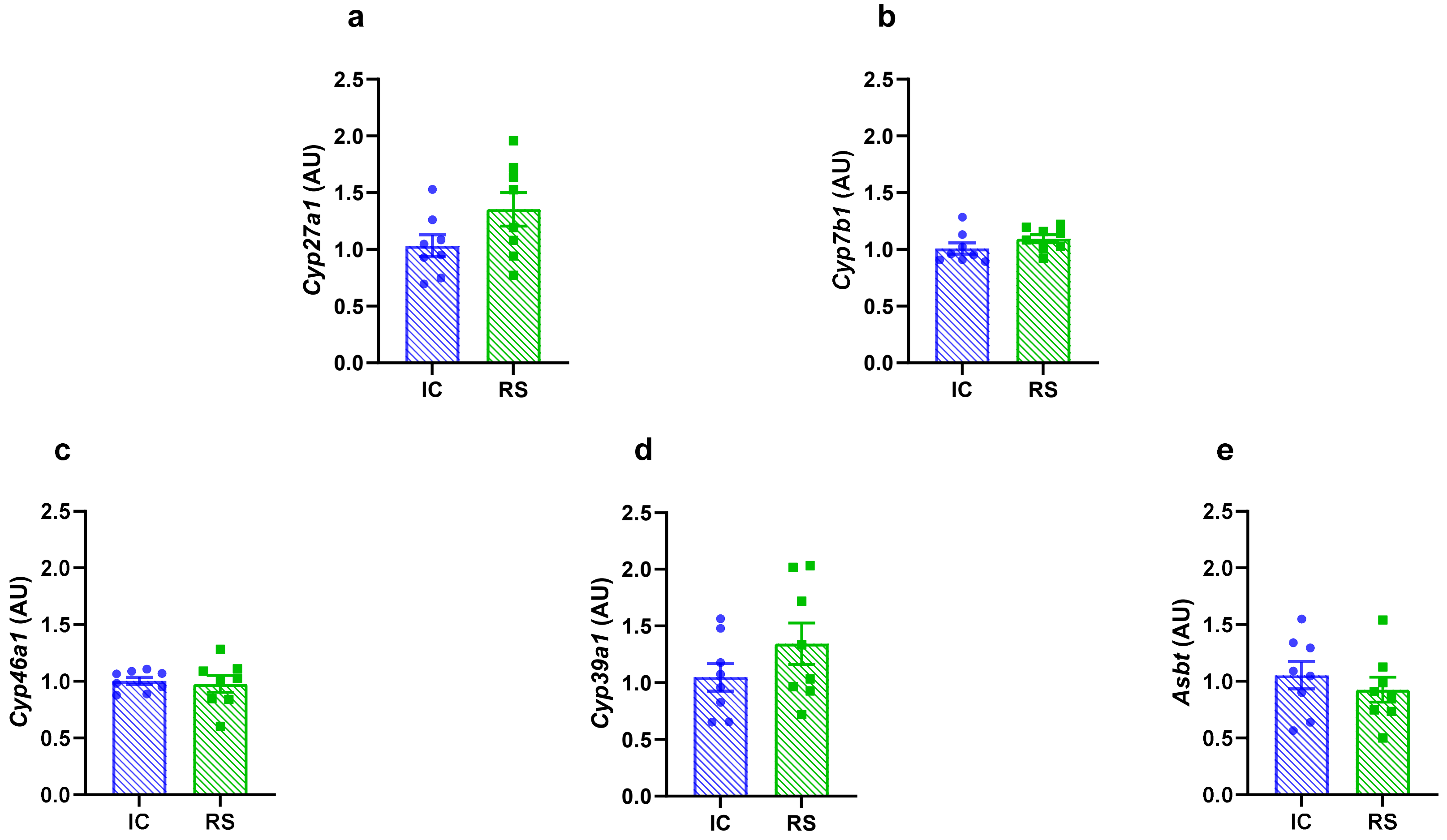
a
b
c
d
e
Cyp27a1 (AU)
Cyp7b1 (AU)
Cyp46a1 (AU)
Cyp39a1 (AU)
Asbt (AU)
2.5
2.0
1.5
1.0
0.5
0.0
IC
RS
